# Coupling and Coordinative Development of Green Finance and Industrial-Structure Optimization in China: Spatial-Temporal Difference and Driving Factors

**DOI:** 10.3390/ijerph191710984

**Published:** 2022-09-02

**Authors:** Lei Nie, Purong Chen, Xiuli Liu, Qinqin Shi, Jing Zhang

**Affiliations:** 1 Cooperative Innovation Center for Transition of Resource-Based Economics, Shanxi University of Finance and Economics, Taiyuan 030006, China; 2 Research Institute of Transition of Resource-Based Economics, Shanxi University of Finance and Economics, Taiyuan 030006, China

**Keywords:** green finance, industrial-structure optimization, coupling coordination, geographic detector

## Abstract

Although the literature has studied the direction and extent of the effect of green finance on industrial-structure optimization, quantitative analysis of the coupling coordination and spatial–temporal differences between green finance and industrial structure is relatively scarce. Therefore, in this paper, we built the theoretical framework of the coupling coordination relationship between green finance and industrial-structure optimization, and then we used the coupling coordination degree and geographic detector model to investigate the spatial–temporal evolution characteristics and influencing factors of the coupling coordination between the two based on the panel data of 31 provinces from 2012 to 2019. The results show that China’s green finance and industrial-structure optimization have basically reached the primary coupling, and the coupling coordination degree is from 0.40 to 0.43, which shows a “W”-type fluctuation trend of recovery. The regional gap of the coupling coordination degree firstly decreased and then increased, showing a change law of “agglomeration, equilibrium and agglomeration”. In the spatial dimension, the high-level coordination region showed an increasing trend of “fragmentation” fluctuation, while the low-level coordination region concentrated in the central and western regions with a tendency of “low value locking”. The trend surface showed a spatial characteristic of “high in the north and low in the south–high in the east and west and low in the middle”. We also found that the dependence of foreign trade and technological innovation are the main factors affecting the coupling coordination degree, and the interaction between government support and human capital synergistic is the crucial channel for the coevolution of green finance and industrial structure to promote green and low-carbon development.

## 1. Introduction

Sustainable development is a key research topic in academia derived from ecological environment deterioration [1,2]; the concept of green, low-carbon, and environmentally friendly development has become a universal consensus in the world. China, as a responsible developing country, has always been committed to achieving peak CO_2_ emissions by 2030 and carbon neutrality by 2060 [3,4]. The goal of “double carbon” marks a new stage of “carbon reduction as the key strategic direction” in China’s ecological civilization construction, which requires changing the traditional industrial system with high energy consumption and high pollution, alleviating the current situation of overcapacity and insufficient innovation, and increasing the investment of green funds to provide a solid foundation for the green and low-carbon transformation of industrial structure [5].

As an innovative mechanism for the financial industry to better serve the transformation and development of green economy [6], green finance can achieve a Pareto-optimal allocation of environmental protection funds and achieve rapid development in China. In 2016, seven ministries and commissions, including the People’s Bank of China, issued the Guidance on Building a Green Financial System [7], constructing a top-level framework system of green finance for the first time. With the continuous improvement in the system, green finance has gradually become an important path and strong support for promoting green and low-carbon development in China [8,9,10]. The development of real industry and capital allocation are corresponding and linked. Promoting the optimization and upgrading of industrial structure is a long-term and complex task in the process of economic and social development of all countries [11]. Timely adjustment of industrial structure is the driver of rapid economic growth, and it has become an inevitable choice for economic development of all countries to promote efficiency through structural coordination [12]. Therefore, as an important starting point for the green and low-carbon transformation of the real economy, the coupling and coordinated development of green finance and industrial-structure optimization have become an important guarantee for realizing the goal of “double carbon”.

So far, many scholars have verified the role of green finance in the optimization and upgrading of industrial structure. On the one hand, most scholars believe that green finance can significantly promote the optimization and upgrading of industrial structure. Agyekum et al. (2021) and Zhang et al. (2021) proposed that green credit and green investment have an important impact on the transformation of industrial structure and can promote the upgrading of industrial structure [13,14]. Furthermore, Wang et al. (2021), based on the mechanism of green finance affecting industrial structure, proposed that green finance can play the role of capital leverage and resource reallocation, thus affecting industrial structure [15]. Similarly, green finance can also act as a regulatory mechanism to affect the effect of renewable-energy-technology innovation on industrial structure upgrading, and only in regions with a high degree of green-finance development can it have a positive promoting effect [16]. On the other hand, some scholars regard green financial policies as restrictive industrial policies, and believe that green-credit policies restrict the financing of energy-intensive enterprises, resulting in inefficient credit allocation and hindering enterprise transformation, which is not conducive to the upgrading of industrial structure [17,18,19]. Therefore, if the green financial and industrial-structure optimization as two separate systems may lead to the result of inconsistent and fuzzy policy, only the two together can allow studying the present situation of coordinated development and influence factors in order to have more realistic significance in the sustainable development of the regional economy and putting forward policy suggestions.

Throughout the past research, many scholars, in view of the green financial and industrial-structure optimization, discussed in this paper form the relatively rich research achievements, but the research conclusions are not completely consistent, and there is a lack of focus on the following issues: (1) Most of the research is limited to the green financial one-way function of industrial structure, mutual coupling, and its quantitative study of the difference of space, which is still in the exploratory stage. Only a few scholars have linked the two, pointing out that green finance is conducive to promoting the coordinated development of the financial industry and the green real industry, which together contribute to the low-carbon transformation of industrial structure and high-quality economic development [20]. (2) As can be seen from the literature review on the measurement methods of green finance and industrial structure in Section 3.1, most scholars have a single measurement index for the two and lack the construction of the index system from a comprehensive perspective. (3) Most of the existing studies conducted theoretical or empirical analysis from the perspective of economics, but did not consider the spatial–temporal characteristics and influencing factors of the coupling coordination degree of green finance and industrial-structure optimization from the perspective of geography.

In view of the above research gaps, based on the panel data of 31 provinces in China from 2012 to 2019, this paper studies the spatial–temporal evolution characteristics and influencing factors of the coupling coordination degree of green finance and industrial-structure optimization in the region, aiming to explore how to enhance the coordination degree and help achieve the goal of “double carbon”. The contribution and innovation of this article may include: (1) In the aspect of research perspectives, unlike previous one-way influence mechanism research, this article combines green financial and industrial-structure optimization into a system from the angle of the coupling relation and the interactive relationship between the two, which has been expanded, further enriching the relationship between the green financial and industrial-structure optimization theory research. (2) In terms of the research content, this paper takes the coupling coordination degree of green finance and industrial structure upgrading as the entry point, focuses more on the study of the evolution of spatial–temporal characteristics, and further investigates its main influencing factors, so as to provide scientific support for the realization of high-level coordination and green and low-carbon development in the region. (3) In terms of the research method, it constructs a theoretical framework for coordinated development of the two, enriches the comprehensive evaluation system of green finance and industrial-structure optimization, and theoretically provides a logical support for the evaluation methods and their interaction of “green finance-industrial-structure optimization” in the region. In addition, in the process of empirical analysis, the entropy method, coupled coordination degree model, and geographic detector model are used to improve the accuracy of the research results. The study of regional differences is of great significance for regional coordinated development.

The rest of this article is arranged as follows. In Section 2, relevant theoretical analysis and hypotheses are presented. Section 3 describes the research methodology and data sources. Section 4 is the analysis and discussion of the research results. Finally, Section 5 provides research conclusions and policy recommendations.

## 2. Theoretical Analysis and Research Hypothesis

**Figure 1:**
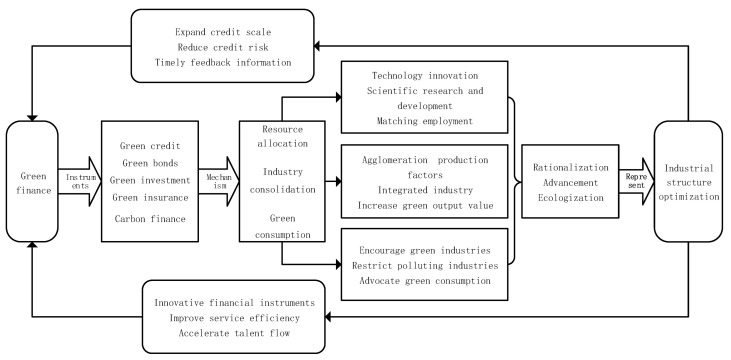
Coupling development path of green finance and industrial structure.

As an emerging financial model, green finance possesses strong economic vitality, which conforms to the new trend in green development in the modern social economy [21]. Therefore, under the condition of promoting the vigorous development of numerous green industries and the adjustment and transformation of traditional industries, industrial-structure optimization will be inevitably promoted [22]. A greater demand for rationalization, upgrading, and ecologization of the industrial structure put forward higher requirements for the service capacity of green finance, so as to promote the improvement in the policy system, financial instruments, and service efficiency of green finance. The financial structure varies with the alteration of industrial structure [23]. Based on the sustainable development theory of economy, externality theory, and Petty Clark theorem, this paper constructs a theoretical analysis framework for the coupling and coordination of green finance and industrial-structure optimization, as shown in Figure 1.

### 2.1. Interaction Theory of Green Finance and Industrial-Institution Optimization

To be specific, for one thing, green finance promotes the transformation of industrial structure to rationalization, advanced, and ecological transformation through three aspects. First of all, green finance can promote the green allocation of capital, provide financial support for energy conservation and environmental protection industries that is in line with the time limit and cost, and encourage scientific and technological R&D and innovation [24,25,26,27]. Secondly, collaborative industrial agglomeration is conducive to reducing redundant construction and carbon consumption, and improving industrial resilience and sustainability [28,29,30]. In order to seek long-term competitive advantages, green enterprises tend to play the cluster effect in areas with strong government support, favorable financial environment and low investment and financing costs [31,32]. Finally, green finance can encourage green consumption by adjusting the consumption structure and create favorable market environment and development conditions for technological innovation of enterprises [33,34,35]. For another, industrial-structure optimization can promote the “re-development” and “re-innovation” of green finance. The development of the green real economy depends on the optimization and upgrading of industrial structure [11]. When the upgrading of industrial structure promotes the improvement in production efficiency, it is necessary to have higher financial supply capacity to adapt to it. If the supply side fails to keep up with this change, it will lead to the mismatch between supply and demand [36]. For financial institutions, the sound development of green enterprises is conducive to their expansion of credit scale, timely feedback of information, reduction in investment risks, improvement in service efficiency and innovation of financial tools, gradually forming a virtuous cycle [37,38,39]. According to the Cord–Clark theorem, the advanced industrial structure will promote the gradual transfer of labor force to the secondary and tertiary industries and provide more innovative talents for the financial market and green enterprises [40]. Based on the above two points, Hypothesis 1 is proposed in this study.

**Hypothesis** **1.** *Green finance and industrial-structure optimization can achieve a benign coupling, and their synergy is conducive to regional green transformation.*

### 2.2. Spatial-Temporal Heterogeneity of the Coupling between Green Finance and Industrial-Structure Optimization

The Green Credit Guidelines issued by China Banking Regulatory Commission in 2012 provide guidance and norms for the banking industry to carry out green credit, aiming to promote the development of green industry through policy guidance. The central bank has also included green-finance performance in the macroprudential assessment (MPA) system, and banks with high green-credit balances will receive extra points in the MPA assessment. Undoubtedly, in the long run, the capital allocation function of green finance plays a positive role in promoting green investment, green productivity, and industrial-structure optimization [41,42,43]; green finance not only restrains the wanton expansion of energy-intensive industries to a certain extent, but also promotes the development of green industries [44,45].

However, in the short term, due to the imperfection of our capital market, the implementation standards of green-credit policy are not clear [46]. In order to complete the task of green credit, banks simply increase the financing constraints of energy-intensive enterprises, which, to some extent, leads to low market efficiency and runs counter to the original intention of rational resource allocation [47]. Green-credit policies have asymmetric effects on the financing of environmentally friendly enterprises and pollution-oriented enterprises [48]. In terms of green enterprise, for science and technology, the research and development cycle is long and investment is high. Green enterprises have not been able to put new technology into production under the premise of rashly made credit and loans more accessible to heavy industry; the threshold can cause heavy industry, in the short term, to not realize capital turnover, and to be unable to complete technological innovation and science and technology research and development, causing stagnation in the industrial structure. Industry has been the leading sector in China for a long time, and high-energy-consumption industry has become the main object of bank loans due to its strong energy advantages and capital base [49]. Therefore, when exploring the path of the green and low-carbon transformation of industrial structure, attention must be paid to high-energy-consumption industries [50,51,52]. With the constant improvement in green financial systems, green finance constantly enriches financial instruments and the broader green-finance main body, highlighting the efficiency of the green industry; the traditional industry has passed the “burst” and “rebirth” stages of its baptism to participate in market competition, with a new gesture to implement green finance in long-term financial and industrial-structure optimization of the benign coupling. Therefore, this paper proposes the following Hypothesis 2.

**Hypothesis** **2.** *The coupling coordination between green finance and industrial-structure optimization has time volatility.*

Due to the differences in water basis, resource endowment and inherent industrial structure of economic development in different regions of China, the development level of green finance in different regions will also be different [53]. In the theory of “pollution paradise Hypothesis”, due to the increase in production cost brought by environmental regulation, highly polluting enterprises will tend to invest and set up factories in areas with weak environmental regulation in order to save costs [54]. Similarly, green finance is essentially a kind of market-oriented environmental regulation, and highly polluting enterprises tend to produce in areas with slow development of green finance [55]. Therefore, Hypothesis 3 is put forward in this paper based on the difference in economic development degree in different regions, the imbalance of environmental regulation intensity, the inconsistency of the development level of green finance and industrial structure, and the immature development degree of the financial market.

**Hypothesis** **3.** *There is regional heterogeneity in the coupling coordination between green finance and industrial-structure optimization.*

## 3. Methodology and Data

### 3.1. Comprehensive Evaluation Index System

Most scholars evaluate the overall development level of green finance in a country or region from the perspective of capital supply. Campelo (2013) believes that the development level of green finance in a country can be measured from the amount of “Equator Principle” added by commercial banks and the balance of green loans [56]. Li (2014) and Clark (2018) measured the development level of green finance of a country from several factors, such as the total number, proportion, and scale of green loans, according to the equator principle commonly used in the banking industry [57,58]. The development time of green finance in China is relatively short, and green credit and green investment are the main forces of green-finance funds. Therefore, some domestic scholars directly use their single index as the measurement index of green finance [59,60]. With the emergence of many green financial products, some scholars have replaced green finance with carbon finance [61]. Combined with existing research [62], this paper establishes a comprehensive evaluation system of green finance to measure green finance through the composite index method based on five dimensions of green credit, green investment, green bonds, green insurance, and carbon finance. In terms of industrial-structure optimization, Kuznets (1957) explained the adjustment and evolution of industrial structure from the factor allocation of the three industries, and believed that the change in industrial structure was a change in the proportion of output value of each industry due to the reallocation of domestic factor resources among various industries [63]. Some scholars also put forward that the optimization and upgrading of industrial structure is to realize the unification of rationalization and advancement in the process of change [64,65], that is, the coordinated development of the industrial structure and the high-quality development of the hierarchy. With the increasingly serious environmental problems, some scholars put forward the ecologicalization of industrial structure on the basis of rationalization and advanced [66], which supplements the optimization and upgrading of industrial structure from the perspective of the ecological environment. Therefore, this paper constructs evaluation indicators from three aspects: rationalization, advanced, and ecological.

Notably, the green-finance index system needs to be elaborated in detail due to its relative complexity. Green credit is characterized by the proportion of green-credit balance and the proportion of interest expenditure in high-energy-consuming industries. The former adopts the green-credit balance of 21 major banking institutions in China, while the latter takes the interest expenditure proportion of industrial enterprises above a designated size as a negative indicator according to the division of six high-energy-consuming industries in the classification of the national economic industry. Green securities select the proportion of the market value of green listed companies in A-shares. The company manually selects the business contents corresponding to the six green industries in the green industry guidance catalogue (2019 edition). A total of 276 green enterprises were finally obtained after removing ST companies and enterprises with missing financial data information. Green investment was measured by the proportion of investment in environmental pollution control and expenditure on energy conservation and environmental protection. Green insurance lacks its statistical data due to a relatively short development period in China. However, agricultural insurance is essentially a means of environmental risk management. Its capability to approximately reflect the situation of green insurance can be attributed to its relative completeness in China. In this paper, the situation of green insurance was reflected by the proportion of agricultural insurance scale and loss ratio. Carbon finance is a kind of low-carbon finance, which is reflected by the proportion of carbon emissions in the loan balance. The smaller the index value, the higher the low-carbon level of financial institutions. In this paper, the range normalization was used to deal with the dimensionless raw index data, and the entropy method was used to determine the weight of each index. The final comprehensive evaluation index system is shown in Table 1.

### 3.2. Coupling Coordination Degree Model

The concept of coupling originates from physics, which refers to the phenomenon that two or more systems unite and promote together through interaction. Coupling is commonly used to reflect the coordinated development of regions or indicators [62]. Although the correlation degree between systems can be reflected by the coupling degree, it is prone to covering up the conclusion of high coordination at a low level. Therefore, it is necessary to introduce the coupling coordination degree model to judge the coordination degree and development degree between systems. This paper refers to the research by Zhang et al. (2022) and the specific model is as follows [67].

(1) $$\left\{ \begin{array}{l} D=\sqrt{C\times T}\\ C=\sqrt{\frac{G_{i}\times I_{i}}{{\left(G_{i}+I_{i}\right)}^{2}}}\\ T=\alpha \times G_{i}+\beta \times I_{i} \end{array}\right.$$

where *D* is the coupling co-scheduling, *C* is the coupling degree, *T* is the comprehensive evaluation index of green finance and industrial-structure optimization, *G_i_* and *I_i_* represent the comprehensive evaluation value of green finance and industrial-structure optimization, respectively, and α and β are the undetermined coefficients. Since green finance and industrial-structure optimization are equally important, both α and β are therefore, assigned to a value of 0.5. The relevant levels of coupling degree and coupling coordination degree are determined through the combination of the existing research and actual situation [52,53,54], as shown in Table 2.

### 3.3. Trend Surface Analysis

Trend surface analysis is a statistical method based on the spatial data. The spatial evolution law and distribution characteristics of a geographical system element can be intuitively reflected using mathematical methods to calculate mathematical surfaces for spatial trend analysis [68]. Taking coupling and co-scheduling as the observation values, the trend surface analysis tool in Arcgis10.2 software was used to simulate the spatial–temporal evolution trend of the coupling coordination degree between green finance and industrial-structure optimization in 31 provinces of China from 2012 to 2019. Let (*x_i_*,*y_i_*) be the spatial location of province *i*, then *Z_i_* (*x_i_*,*y_i_*) is the trend function of province *i*, where *X*-axis and *Y*-axis represent the east–west and north–south directions, respectively.

### 3.4. Geographical Detector

A geographic detector is a statistical tool to detect spatial differentiation and spatial interaction to reveal the “factor force” behind it. Using geographical detectors, the driving factors of the coupling coordination degree between green finance and industrial-structure optimization were analyzed in this paper, as well as the interaction between the driving factors. The formula is as follows [69].

(2) $$q=1-\frac{1}{n\sigma_{}^{2}}\sum_{i=1}^{m}n_{i}\sigma_{i}^{2}$$

Among them, *q* is the detection force index of the influencing factor of the coupling coordination degree, which reflects the interpretation degree of the spatial differentiation of this attribute by the detection factor; n and $\sigma_{}^{2}$ represent the number and variance of samples in the whole study area; *m* is the type of driving factor; *n_i_* and $\sigma_{i}^{2}$ represent the number and variance of class *i* factor samples; and the value range of *q* is between 0 and 1. The closer it is to 1, the greater the influence of this factor on the coupling coordination degree, and vice versa.

Interaction detection can evaluate the influence of the interaction between different risk factors on the coupling co-scheduling. The interaction relationship of two factors can be divided into the following categories, as shown in Table 3.

Financial development will change with economic development, trade openness, and other factors [70]. In addition, the coupling coordination degree of green finance and industrial-structure optimization will also be affected by economic development, educational innovation, social environment, government policies, and other aspects. Therefore, referring to the existing literature [15], human capital (huca), technological innovation (tein), environmental regulation (enre), fixed-asset investment (fixi), foreign-trade dependence (export), urbanization rate (ur), foreign direct investment (fdi), and government support (gov) were taken as the influencing factors. The influencing factors and definitions are shown in Table 4.

### 3.5. Data

The green-finance data came from the report on the social responsibility of the banking industry, the statistical table of green credit of 21 major banks disclosed by the CBRC, the statistical yearbook of China’s industrial economy, the statistical yearbook of China’s environment, the China Insurance Yearbook, the CSMAR database, and the CDEAS database. The data of economic and social indicators involved in this paper were from the China Statistical Yearbook, China Social Statistical Yearbook and China Energy Statistical Yearbook.

## 4. Empirical Results

### 4.1. Time Evolution Characteristics

#### 4.1.1. China’s Overall and Regional Coupling Coordination Degree Shows a “W” Shaped Fluctuation and Upward Trend

**Figure 2:**
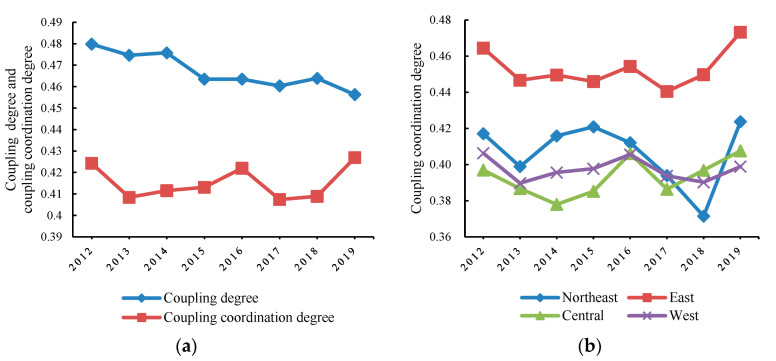
Schematic diagram of annual variation trend of national and regional coupling coordination.

The coupling degree and coupling coordination scheduling of China in each year were calculated through the coupling coordination degree model. In addition, the coupling development of varied regions was further reflected according to the division of four major economic regions in China. The results were sorted according to the average value, as shown in Table 5 and Figure 2.

From 2012 to 2019, a “W” trend of fluctuation and recovery could be seen in the coupling coordination degree of green finance and industrial-structure optimization in China and various regions, indicating an on-going, benign coordinated development amid fluctuations in the coordinated development of green finance and industrial-structure optimization in China. The average coupling coordination degree was 0.415, indicating that China has achieved primary coordination on the whole, and green finance and industrial-structure optimization have achieved preliminary coupling development on the whole. The average coupling degree was 0.467, which was in the primary-coupling state; however, it decreased in each of the past years, indicating an annually downward trend in the correlation degree between the two. From the perspective of time change, the coupling coordination degree showed a trend of “decline (2012–2013) → rise (2013–2016) → decline (2016–2017) → rise (2017–2019)”, which may be attributed to the financing difficulties of industrial enterprises caused by the unclear implementation standards of regional green-credit policies [19]. After the release of the guidelines, the co-scheduling fell by a year due to the lagging effect of the policies. However, under the influence of government support and guidance, financial institutions, green enterprises, and other microentities’ investment and financing, coupled collaborative scheduling rebounded slightly. This rebound was unsustainable due to the limited policy strength and uncompleted green financial system. The coordination degree will not continue to rise before the adjustment and recovery of microeconomic entities. The growth rate was obvious in 2019, with the highest value of 0.43, indicating that green finance and industrial-structure adjustment have gradually realized benign interaction with the strengthening of China’s ecological civilization construction and the orderly promotion of green development. Therefore, in the short term, the coupling development trend of green finance and industrial-structure optimization is fluctuating downward, but in the long-term interaction and integration, the two can achieve benign coupling. Hypotheses 1 and 2 are verified.

From a regional perspective, it has formed a situation in which the east takes the lead, the northeast rapidly catches up, the central region continues to develop, and the western region transits steadily. The change in regional order was relatively stable from 2012 to 2017, and the rest of the region fluctuated greatly from 2017 to 2019, except for the eastern region. The specific performance is as follows: the northeast showed an obvious “V” change, and the ranking order was changed to 2 → 4 → 2. The central region increased by 3% for two consecutive years, and the order changed to 4 → 2 → 3, with a growth rate higher than that of the western region, which remained relatively stable. The order changing to 2 → 3 → 4 was related to the development strategies of the four regions. Zhang et al. (2022) studied the coupling coordination relationship between green finance and environmental performance, and concluded that the spatial distribution of coupling coordination degree is not random, but closely related to regional economic development, government regulation, investment conditions, etc. From the perspective of the spatial pattern, the eastern region has significant advantages [67]. Similarly, in the study of this paper, the strong economic and technological foundation and sound financial market in the eastern region are conducive to the realization of the cumulative circular effect of benign coordination between green finance and industrial-structure optimization. Northeast China has long relied on heavy industry, which, therefore, has great resistance to industrial-structure adjustment. In recent years, with the promotion and implementation of a series of major decisions and arrangements, local governments have accelerated the adjustment of industrial structure and the reform of state-owned enterprises. There exists an obvious growth rate of green finance, as well as an enhanced interaction with industrial-structure optimization. Despite the favorable ecological and resource advantages in the central and western regions, its development speed remains relatively slow due to the short development time of green finance, the shortage of funds and technology, and the imperfect relevant-policy system of financial institutions. In conclusion, Hypothesis 3 is proved.

#### 4.1.2. The Regional Gap of Coupling Coordination Degree Tends to Narrow First and then Expand

**Figure 3:**
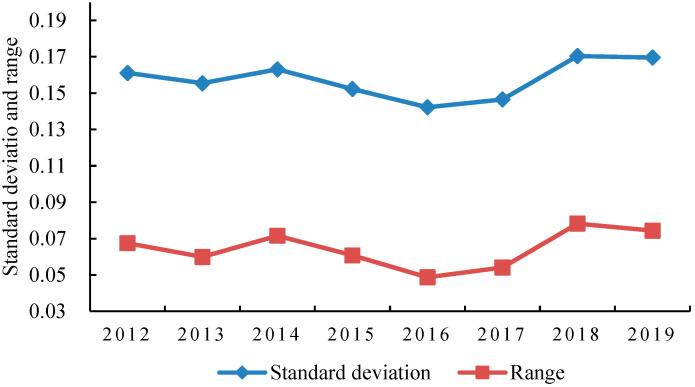
Annual variation trend chart of regional coupling coordination degree difference.

In order to further analyze the regional differences in the coordinated scheduling of green finance and industrial-structure optimization in various regions of China, the annual intervals and standard deviations of the four regions were calculated (Table 5 and Figure 3).

From the perspective of the regional gap, the interval and standard deviation showed a trend of first decreasing and then increasing, reflecting that the coupling and coordination of various regions were “agglomeration → equilibrium → agglomeration”. Cao et al. (2022) believe that a good financial structure can provide power for an economic operation, effectively attract capital inflows to increase regional investment opportunities, form a “capital agglomeration” effect, and help realize a virtuous cycle of economic development [71]. From 2012 to 2016, the regional coupling coordination degree decreased from 0.067 to 0.049, and the standard deviation also decreased from 0.161 to 0.142, indicating that the coordination of green finance and industrial-structure optimization in various regions transitioned from agglomeration to equilibrium, the regional gap weakened, and most regions were still in the primary coordination stage. From 2016 to 2019, the range and standard deviation increased to 0.074 and 0.170, respectively, and the coupling coordination values tended to be scattered. The emergence of higher-level-coordination provinces widened the regional gap, which may be attributed to the different financial and industrial foundation of each region, as well as the large gap in regional coupling coordination at the beginning of the guidelines. With the gradual promotion of green-finance policy and the continuous upgrading of industrial structure, the low-level-coupling regions have been preliminarily developed, while the high-level-coupling-coordination degree was relatively stable at this stage, always remaining between 0.44 and 0.45, and the regional gap gradually narrowed. In the latter stage, the local market effect remained relatively strong, and the northeast, central, and western regions fluctuated greatly with the significant improvement in the coupling coordination degree in the eastern region, reflecting the difference between the coupling development of green finance and industrial-structure optimization.

### 4.2. Spatial Evolution Characteristics

**Figure 4:**
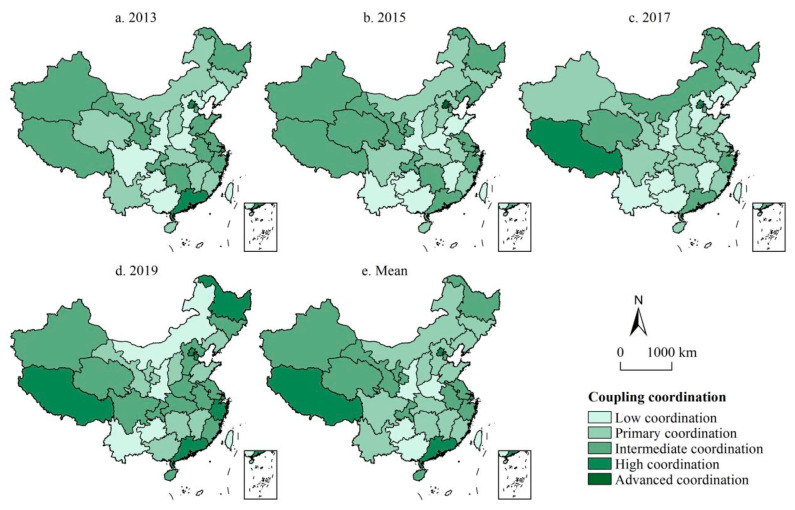
Temporal and spatial distribution of coupling coordination degree of provinces and regions in China, 2013–2019.

In order to reflect the spatial changes in coupling and coordination between green finance and industrial-structure optimization in various regions of the country, the coupling and coordination scheduling in 2013, 2015, 2017, and 2019 were selected, respectively, and the time–space distribution map of the national coupling and coordination degree was drawn (Figure 4).

#### 4.2.1. The Number of High-Level-Coordination Areas Fluctuated and Increased, Showing the Distribution Characteristics of “Fragmentation”

High-level coupling and coordination are composed of high-level and high-quality coordination. According to Figure 4, the number of provinces with high-level coupling and coordination of green finance and industrial-structure optimization in China showed a fluctuating upward trend, but the distribution was relatively scattered, showing a “fragmented” distribution characteristic dominated by the east. This is similar to the research conclusion that economic centers such as Beijing and Shanghai are more conducive to the coupling and coordination of economic factors [71]. In terms of the change in the number of provinces, the number of high-level-coupling and -coordination provinces was changed to 3 (2013) → 2 (2015) → 3 (2017) → 7 (2019), and the average value of the coupling and coordination degree corresponding to the second level also showed a fluctuating upward trend of 0.408 (2013) → 0.413 (2015) → 0.407 (2017) → 0.427 (2019). From the perspective of regional distribution, from 2012 to 2018, the high-level-coordination areas remained relatively stable in Beijing, Shanghai, eastern Guangdong, and western Tibet. The eastern region showed the convergence characteristics of “clubs” gathering in patches. Due to the proximity of geographical location, regions with similar economic foundation and industrial structure are more likely to achieve steady economic convergence [72]. By 2019, new Tianjin, Heilongjiang, and Zhejiang were still concentrated in the eastern region, indicating the role of the eastern region as a high-level agglomeration and development frontier area with the coupling and coordination of green finance and industrial-structure optimization in China. The three major growth poles, with Beijing, Shanghai, and Guangdong as the mainstays, have facilitated the coordinated development of interprovincial connected regions, while individual regions in the west and northeast have also transitioned to a high-level coupling and coordination stage due to their ecological or industrial development advantages, which is conducive to radiating and driving the coordinated development of the surrounding low-level-coupling areas.

#### 4.2.2. Low-Level-Coordination Areas Tend to Be “Low Value Locked”, Concentrated in the Central and Western Regions

As can be seen from Figure 4, the spatial pattern of China’s coupling coordination degree remained relatively stable, the coupling coordination between the east and the west and the northeast remained favorable, and the coupling coordination degree in the central region was always lagging behind, with the characteristics of “low value locking”. Corresponding to the high level, the low-level coupling coordination includes low-level and primary coordination. From the perspective of the change in the number of provinces, the number of low-level-coupling-coordination provinces was changed to 16 (2013) → 16 (2015) → 22 (2017) → 12 (2019). Compared with 2013, the change in 2015 was small, and the low-level-coordination areas tended to relatively move southward. By 2017, the low-level-coordination areas accounted for approximately 3/4 of the provinces in China, concentrated in the vast area centered on the central part, achieving low-level balanced coordination on the whole and narrowing the gap in the degree of coordination between regions. By 2019, except for Inner Mongolia, Shaanxi, Guizhou, and Yunnan, which are still in low-level coordination, other provinces achieved the above-mentioned primary coordination, and the regional gap had significantly widened. On average, among the 16 low-level-coordinated regions such as Guizhou, Shaanxi, Guangxi, and Henan, 11 belong to the central and western regions. It can be seen that the coupling and coordinated development of green finance and industrial-structure optimization in the central and western regions is relatively backward, and the “trickle-down effect” on the surrounding regions remains weak, which may be related to the relatively backward industrial level and the lack of active financial markets and policy influence in these regions [73].

#### 4.2.3. The Coupling Coordination Degree Presents a Spatial Pattern of “High in the North and Low in the South-Low between High Schools in the East and West”

**Figure 5:**
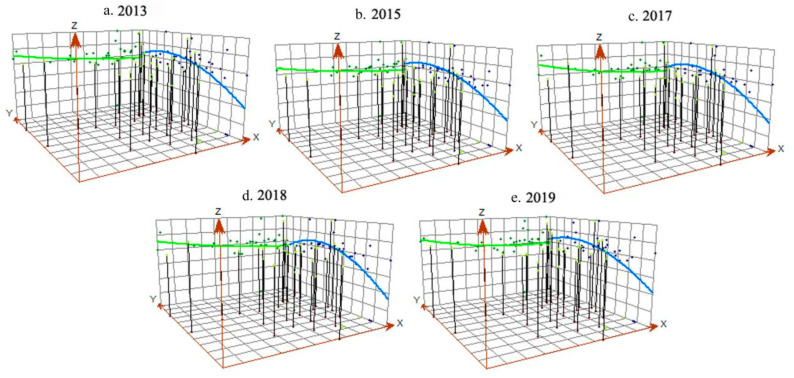
Trend surface analysis of coupling coordination degree, 2013–2019.

Combined with the trend surface analysis (Figure 5), the coupling coordination degree of China’s green finance and industrial-structure optimization generally presents a distribution trend of “high in the North and low in the South–low between East and West High Schools” in the spatial pattern. In the north–south direction (*Y*-axis), the interaction between green finance and industrial-structure system in the northern region remains relatively stable, which is always higher than that in the southern region. Compared with the previous three years, the degree of coupling and coordination in the southern region increased rapidly in 2019, about twice that in 2017. On the whole, the spatial gap between the north and the south gradually narrowed, showing a balanced development trend. In the east–west direction (*X*-axis), there has always been a positive “U” type distribution between senior high schools and junior high schools. However, the eastern region is slightly higher than the western region, and the transition trend to the eastern and western region is basically the same, indicating that the interaction between green finance and industrial-structure optimization in the western region has gradually deepened, and the gap between the western and eastern regions has gradually narrowed. However, there remains a low coupling coordination degree and a weak change, as well as a lagging coupling and coordinated development of green finance and industrial-structure optimization in the central region.

### 4.3. Analysis on the Influencing Factors of the Coupling and Coordination between Green Finance and Industrial-Structure Optimization

#### 4.3.1. Analysis of Main Influencing Factors

In this paper, the coupling co-scheduling (*D*) of green finance and industrial-structure optimization was taken as the dependent variable, and the above-described eight factors were taken as the dependent variable, which were discretized through the natural breakpoint method, and divided into type variables. Based on the geographic detector model, the influence of different factors in each period on the coupling coordination degree of green finance and industrial-structure optimization can be obtained, which is expressed by *q* value, and the time change trend chart of *q* value of each risk factor can be drawn. See Table 6 for details.

As illustrated in Table 4, the influence degree varied with different factors according to the influence of each factor on the coupling coordination degree of green finance and industrial-structure optimization (Q value). According to the ranking of the top five factors that have the greatest impact on the coupling coordination degree throughout the year, foreign-trade dependence (0.611) > technological innovation (0.562) > fixed-asset investment (0.423) > urbanization (0.391) > environmental regulation (0.339), which can be identified as the main factors affecting the coupling coordination between green finance and industrial-structure optimization. Some scholars have concluded that foreign trade can not only provide a large amount of capital demand for green finance, but also promote the interaction and coordination between green finance and green industry by influencing the energy structure [74,75]. The influence of technological innovation is growing rapidly, suggesting that the technological research and development of higher talents can also serve as an important factor to realize the coordinated development of green finance and industrial-structure optimization on the basis of strengthening the green concept in higher education [72]. Fixed-asset investment is the most important part to connect the utilization of money and physical investment of enterprises, mainly to used funds to construct the physical assets. Whether enterprises can effectively use capital to carry out the physical construction of green industry figures predominantly in the coordinated development of green finance and industrial structure [76], which reflects the increasing role of urban-construction investment in the coordinated development of green finance and industrial-structure optimization. Urbanization is the cause and driving force of industrial diversification. The increase in urban population not only brings more green capital investment and consumption demand and promotes the perfect development of green enterprises and related financial institutions, but also helps to optimize the employment structure and the efficiency of resource allocation [77], and promotes the transformation of industrial structure from traditional industries to tertiary industries and high-tech industries. Environmental regulation is a negative index reflecting the emission intensity of industrial “three wastes”. As a negative indicator, environmental regulation reflects the emission intensity of industrial “three wastes” [78]. When the environmental-regulation index drops, it means that industrial enterprises have reduced the emission of pollutants. This process is the embodiment of green finance forcing polluting enterprises to reduce environmental pollution through technological innovation and realize the upgrading of industrial structure.

From the perspective of time change, except for the downward trend in urbanization rate during 2012–2015, the other four main factors showed a fluctuating upward trend. The main influencing factors in 2012 are ranked as follows: fixed-asset investment (0.724) > foreign-trade dependence (0.684) > urbanization (0.607) > technological innovation (0.502) > environmental regulation; while in 2015, they are ranked as follows: fixed-asset investment (0.820) > technological innovation (0.735) > foreign-trade dependence (0.712) > urbanization (0.529) > environmental regulation (0.323). There exists an increased impact of technological innovation on the coupling and coordination of green finance and industrial structure. From 2016 to 2019, the fluctuations of all factors weakened, showing a general trend of decreasing first and then increasing, which may be related to the impact of the international market downturn in 2015, the overall weakness of external demand, and trade friction. In addition, the improvement in China’s own green financial system and the technological-innovation ability of enterprises have also been improved, which, to some extent, reduces the participation of imports and exports in the impact of green finance and industrial structure. The order of the main influencing factors has developed from technological innovation (0.682) > foreign-trade dependence (0.615) > fixed-asset investment (0.438) > urbanization (0.436) > environmental regulation (0.432) in 2016 to foreign-trade dependence (0.625) > technological innovation (0.584) > environmental regulation (0.416) > urbanization (0.403) > fixed-asset investment (0.322) in 2019. The importance of foreign trade has become increasingly prominent. Some scholars have found that foreign trade, as the main driver of green finance, is a hot word in academic research [79]. With the evolution of time, it can be seen that foreign trade and scientific and technological innovation have always maintained a high influence, and the influence gap between fixed-asset investment, urbanization, and environmental regulation tends to narrow, indicating that the factors affecting the coordinated development of green finance and industrial-structure optimization are gradually turning to diversification. Among them, the impact of environmental regulation has gradually increased, reflecting the increasing importance of government regulation on the degree of coupling coordination.

#### 4.3.2. Interaction Detection and Analysis

Geographical detectors were used to further analyze the interaction between different risk factors, and evaluate whether each factor affects the coupling and co-scheduling of green finance and industrial-structure optimization through interaction. The results are shown in Table 7.

The *q* value on the diagonal in the above table refers to the value of each factor acting independently, and the *q* value below the diagonal represents the *q* value obtained after the factor interacts with other factors. It can be seen that the *q* value below the diagonal is much higher than that of the self-independent effect, indicating that the interaction effect is larger than the self-independent effect. According to the detection type of two-factor interaction, the interaction between government support and any other factors is nonlinearly enhanced, showing that the government features in the coupling and coordination of green finance and industrial-structure optimization. Government guidance provides clear development objectives, policy support, and capital supply for regional green finance and industrial-structure optimization with a “visible hand”. Government intervention plays an important role in green investment and can significantly promote green economic performance under the role of green finance [80]. The interaction between human capital and environmental regulation, fixed-asset investment, foreign-trade dependence, foreign direct investment, and government support is nonlinear. The reason is that the widespread popularization of higher education will not only improve the environmental awareness of residents in the new era, but also enhance the concept of green consumption and investment, thereby providing demand support for the development and production of green enterprises. In addition, the education level of the region directly affects the cultivation of local, high-skilled talents and the improvement in urbanization level, which is consistent with the “big market” trend in foreign direct investment and is conducive to promoting the upgrading of industrial structure. In a word, the interaction of these factors exceeds the accumulation of their individual effects, indicating that under the joint action, all the influencing factors have a significant role in improving the coupling coordination between green finance and industrial-structure optimization.

## 5. Conclusions and Suggestions

### 5.1. Conclusions

In this paper, according to the relevant panel data of 31 provinces, municipalities, and autonomous regions in China from 2012 to 2019, the coupling coordination model was applied to measure the coupling coordination between green finance and industrial-structure optimization, and the spatial–temporal characteristics and regional differences of the coupling coordination degree were investigated on this basis. In addition, the influencing factors of the coupling coordination were analyzed using the geographic detector model. The conclusions are as follows:

(1) In terms of time, green finance and industrial-structure optimization in China and various regions basically achieved primary coupling and coordination during the research period, showing a “W”-type fluctuation recovery trend, and moving towards a higher-level benign coordination in the fluctuation. There might be short-term volatility and downside risk, but in the long run, the direction was towards benign coordination. This is similar to the research results of Zhang et al. (2022) [67]. The order fluctuation between regions was concentrated in the midwest, forming a situation in which the east took the lead, the northeast quickly caught up, the central region continued to develop, and the western region transited steadily. It can be seen that with the development and improvement in green finance, it achieved initial industrial integration with various regions, broke through the bottleneck of coupling development, and moved to the right track. The regional gap of coupling coordination degree first narrowed and then expanded, showing the change law of “agglomeration → equilibrium → agglomeration”. The difference in coupling development between green finance and industrial-structure optimization was gradually reflected. It will bring a new round of coupled development opportunities. In the next stage, most provinces can bring a radiation-driving effect to the surrounding areas on the basis of realizing self-development [74].
(2) From the perspective of spatial evolution, during the study period, the number of provinces with high-level coupling and coordination degree between green finance and industrial-structure optimization in China fluctuated and increased, but the distribution was relatively scattered, showing a “fragmented” distribution characteristic dominated by the east. The three growth poles dominated by Beijing, Shanghai, and Guangzhou made the east a high-level-agglomeration and -development frontier area of coupling and coordination between green finance and industrial-structure optimization in China. However, the coupling coordination degree of the central region always lagged behind, with the characteristics of “low value locking”, and the same is true for the coupling and coordinated development of green finance and industrial-structure optimization. Yang et al. (2022) found, in their study on the coupling and coordination relationship between sustainable development and ecosystems in Shanxi Province, that due to the existence of the “resource curse”, the economic and ecological coordination of resource-based provinces is facing severe challenges [81]. Central China and Northeast China are rich in energy, and the traditional industrial model may not be able to achieve a good coupling with the emerging green finance in a short period of time. The central region can refer to the development model of Northeast China to realize its own green coordination. In terms of spatial pattern, the coupling coordination degree generally presents a distribution trend of “high in the north and low in the South–low between high schools in the East and west”, and the regional gap in the east–west direction is larger than that in the north–south direction.
(3) Foreign-trade dependence, technological innovation, fixed-asset investment, urbanization, and environmental regulation serve as the main influencing factors. With the evolution of time, the influence of foreign trade and science and technology innovation always stayed high, and the impact gap between fixed-asset investment, urbanization, and environmental regulation had a narrowing trend, indicating that the factors affecting the coupling and coordinated development of green finance and industrial-structure optimization are gradually changing into diversity, which gradually increases the influence of environmental regulation, reflecting the increasingly important role of government monitoring for coordination. Many scholars have empirically analyzed the positive role of environmental regulation in green technology innovation and financial development, and believe that environmental regulation is an important prerequisite for sustainable development [82,83,84]. In the detection and analysis of interaction items, government support and human capital show strong interaction ability, indicating that in the process of development, attention should be paid to the interaction between various factors to better promote the coordinated development of green finance and industrial-structure optimization.

### 5.2. Suggestions

Based on the research on the spatial–temporal evolution characteristics and influencing factors of the coupling and coordination between green finance and industrial-structure optimization in China, the following suggestions are put forward for the coordinated development of green finance and industrial institutions in the future to jointly facilitate low-carbon transformation, and achieve the goal of “double carbon”:

(1) Under the guidance of government policies, the coordination effect between green finance and regional industrial structure in China is more obvious. Therefore, we should further play the leading and exemplary role of policies. First of all, the Chinese government should take the goal of “double carbon” as an opportunity to clarify the role of various green financial instruments in energy conservation, emission reduction, clean production, and other aspects, and clarify the responsibilities of relevant subjects, so as to promote the coordinated development of green finance and industrial structure. The EU carbon financial market is a representative carbon financial market in the world. Its emission-trading scheme sets different emission and trading standards for gases in specific fields, which is of certain reference significance for China, which has established the pilot carbon-emission-trading scheme at the beginning. Second, an inclusive green-technology revolution should be encouraged [85] to expand the regional scope of green-finance implementation from point to point. With reference to the UK, Germany, Australia, and other countries, national investment banks are set up to promote private and social investment in order to solve the problem of financing barriers for low-carbon projects [86]. For regions in urgent need of industrial-structure transformation, it is necessary to strengthen the support for local green enterprises and green-credit issuance. Finally, we should further improve the green-financial-market system, accelerating the cross-domain flow between green financial instruments. Intensifying green-credit support in the west and central regions, and green enterprises are encouraged to settle in. Optimizing the utilization and allocation of funds, guide green funds towards construction projects of energy-saving and environmental-protection enterprises to achieve a local market effect [87].
(2) The differentiated development mode should be implemented, and the development direction should be focused on according to the coupling and coordination stage of green finance and industrial-structure optimization. The coupling conditions in the eastern region are better, so the product innovation and risk management of green finance should be strengthened. The United States pays attention to the innovation of green financial products. For example, in the aspect of green credit, the United States has launched unsecured preferential loans to support the development of fuel-saving technology, and carried out personalized insurance design in the aspect of green insurance, which gives certain enlightenment for the innovation of a green-finance model in the east. Northeast regions in China, which have long relied on traditional industries for development, are facing the “low development trap” of green finance and industrial-structure optimization. We should strengthen the transformation and upgrading of traditional industrial chains, pay attention to the flexible use of green funds, and give play to the unity and regularity of their development steps. As the central region lags behind in the development of primary green finance, it should focus on the supporting role of industrial-structure optimization in green finance. The western region is faced with the problem that the industrial-structure optimization lags behind; therefore, we should give priority to the supporting role of green finance in the industrial-structure optimization. Existing studies have verified the positive role of green finance in achieving carbon neutrality based on the implementation of renewable-energy emission reduction policies [88]. Shanxi, Shaanxi, Inner Mongolia, Xinjiang, and other energy agglomeration provinces should make full use of their energy advantages and play the role of green finance in funding the development of renewable energy.
(3) We should give full play to factors such as dependence on foreign trade and technological innovation, and strengthen the interaction between government support and human capital and other factors. The implementation of China’s “neighborhood” policy to strengthen the economic and trade exchanges and cooperation with other countries; in 2020, China’s import and export of countries along the “area” totaled more than CNY 9 trillion, which accounts for large infrastructure investment, so the grave environmental protection pressure needs to provide financial support to green buildings under green financial standards. In addition, we should also bring into play the regulatory role of local governments, regulate pollutant emissions by enterprises, encourage enterprises to step up technological innovation in energy conservation and emission reduction projects, and enhance their sense of social responsibility. In the process of development, attention should be paid to the interaction between various factors so as to better promote the coordinated development of green finance and industrial-structure optimization.

## Figures and Tables

**Table 1:** Comprehensive evaluation index system.

Target Layer	Primary Index	Secondary Index	Index Definition	Effect	**Weight**
Green finance	Green credit	Proportion of green-credit balance	Green-credit balance/GDP	Positive	0.123
		Proportion of interest expense of high-energy-consuming industries	Interest expenditure of six high-energy-consuming industries/total interest expenditure of industries above designated size	Negative	0.050
	Green securities	Proportion of market value of green enterprises	Market value of green enterprises/total market value of shares	Positive	0.316
	Green investment	Proportion of investment in environmental pollution control	Investment in environmental pollution control/GDP	Positive	0.104
		Proportion of energy conservation and environmental protection expenditure	Energy conservation and environmental protection expenditure/local general-public budget expenditure	Positive	0.080
	Green insurance	Proportion of agricultural insurance scale	Agricultural insurance compensation/total property insurance compensation	Positive	0.213
		Agricultural insurance loss ratio	Agricultural insurance compensation/agricultural insurance income	Positive	0.093
	Carbon finance	Financial carbon intensity	Carbon emissions/loan balance	Negative	0.022
Industrial structure optimization	Rationalization	Coordination degree of industrial development	Theil index	Positive	0.146
	Advanced	Advanced degree of industrial development	Improved Moore index	Positive	0.397
	Ecologicalization	GDP energy intensity	Total energy consumption/GDP	Negative	0.144
		Air pollution	SO_2_ emission in industrial waste gas	Negative	0.210
		Sewage disposal	Urban-sewage treatment rate	Positive	0.103

**Table 2:** Classification of coupling degree and coupling coordination degree.

Coupling Degree	Coupling Level	Coupling Coordination	Coordination Level
0 < C < 0.42	Basic coupling	0 < D < 0.37	Low coordination
0.42 ≤ C < 0.45	Low-level coupling	0.37 ≤ D < 0.41	Primary coordination
0.45 ≤ C < 0.47	Primary coupling	0.41 ≤ D < 0.47	Intermediate coordination
0.47 ≤ C < 0.48	Intermediate coupling	0.47 ≤ D < 0.6	High coordination
0.48 ≤ C < 1	High coupling	0.6 ≤ D < 1	Advanced coordination

**Table 3:** Detection types of two-factor interactions.

Basis	Interaction
q(X1∩X2) < Min(q(X1), q(X2))	Nonlinear weakening
Min(q(X1), q(X2)) < q(X1∩X2) < Max(q(X1), q(X2))	Single-factor nonlinear weakening
q(X1∩X2) > Max(q(X1), q(X2))	Two-factor enhancement
q(X1∩X2) = q(X1) + q(X2)	Independent
q(X1∩X2) > q(X1) + q(X2)	Nonlinear enhancement

**Table 4:** Influencing factors and definitions.

Influencing Factors	Definition
Huca	Proportion of college students in the total number of the region
Tein	Proportion of internal expenditure of regional research and experimental development (R&D) funds in regional GDP
Enre	Environmental-regulation index (the evaluation values of industrial wastewater, sulfur dioxide, and smoke emissions)
Fixi	Proportion of fixed-asset investment in regional GDP
Export	Proportion of the total import and export of the region in regional GDP
Ur	Proportion of urban population in the total population
Fdi	Proportion of actual investment in regional GDP
Gov	Proportion of government budget expenditure in regional GDP

**Table 5:** Summary of coupling degree and coupling coordination degree.

Index	Region	2012	2013	2014	2015	2016	2017	2018	2019	Mean	Rank
Coupling degree	East	0.477	0.468	0.468	0.453	0.458	0.449	0.460	0.452	0.461	4
	Central	0.488	0.485	0.477	0.458	0.465	0.458	0.458	0.437	0.466	3
	West	0.475	0.476	0.477	0.471	0.468	0.470	0.474	0.468	0.472	1
	Northeast	0.495	0.470	0.494	0.480	0.459	0.465	0.447	0.461	0.471	2
	China	0.480	0.475	0.476	0.463	0.463	0.460	0.464	0.456	0.467	
Coupling coordination	East	0.464	0.447	0.449	0.446	0.454	0.440	0.450	0.473	0.453	1
	Central	0.397	0.387	0.378	0.385	0.406	0.386	0.397	0.408	0.393	4
	West	0.406	0.390	0.396	0.398	0.406	0.394	0.390	0.399	0.397	3
	Northeast	0.417	0.399	0.416	0.421	0.412	0.394	0.371	0.424	0.407	2
	China	0.424	0.408	0.412	0.413	0.422	0.407	0.409	0.427	0.415	
	Range	0.067	0.060	0.072	0.061	0.049	0.054	0.078	0.074	0.067	
	Standard deviation	0.161	0.155	0.163	0.152	0.142	0.147	0.170	0.170	0.161	

**Table 6:** Annual change in risk factor Q value.

Year	Huca	Tein	Enre	Fixi	Export	Ur	Fdi	Gov
2012	0.288	0.502	0.255	0.724	0.684	0.607	0.160	0.076
2013	0.115	0.591	0.228	0.583	0.682	0.611	0.122	0.076
2014	0.146	0.592	0.361	0.683	0.667	0.588	0.223	0.053
2015	0.139	0.735	0.323	0.820	0.712	0.529	0.289	0.057
2016	0.188	0.682	0.432	0.438	0.615	0.436	0.265	0.061
2017	0.166	0.602	0.476	0.360	0.588	0.447	0.382	0.162
2018	0.085	0.614	0.385	0.260	0.586	0.373	0.323	0.118
2019	0.282	0.584	0.416	0.322	0.625	0.403	0.158	0.039
Whole	0.121	0.562	0.339	0.423	0.611	0.391	0.140	0.048
*p* value	0.460	0.000	0.000	0.000	0.000	0.000	0.016	0.999

**Table 7:** Interaction results of two factors.

	Huca	Tein	Enre	Fixi	Export	Ur	Fdi	Gov
Huca	0.121							
Tein	0.655 *	0.562						
Enre	0.476 **	0.701 *	0.339					
Fixi	0.583 **	0.630 *	0.716 *	0.423				
Export	0.745 **	0.686 *	0.758 *	0.685 *	0.611			
Ur	0.487 *	0.629 *	0.591 *	0.536 *	0.640 *	0.391		
Fdi	0.370 **	0.633 *	0.481 *	0.624 **	0.680 *	0.529 *	0.140	
Gov	0.210 **	0.663 **	0.595 **	0.516 **	0.770 **	0.572 **	0.265 **	0.048

Note: “*”, “**” represents two-factor enhancement and nonlinear enhancement, respectively.

## Data Availability

The green credit data are from the statistical table of green credit of 21 major banks in China and the report on social responsibility of the banking industry disclosed by the CBRC. The green investment data comes from the China Statistical Yearbook, the green securities data comes from the CSMAR database, the green insurance data comes from the China Insurance statistical yearbook, and the carbon finance data comes from the CDEAS database. In addition, the economic and social indicators involved in this paper can be obtained from China Industrial Statistics Yearbook, China Environmental Statistics Yearbook, China Social Statistics Yearbook and China Energy Statistics Yearbook.
